# Effect of Aging, Gender and Sensory Stimulation of TRPV1 Receptors with Capsaicin on Spontaneous Swallowing Frequency in Patients with Oropharyngeal Dysphagia: A Proof-of-Concept Study

**DOI:** 10.3390/diagnostics11030461

**Published:** 2021-03-07

**Authors:** Weslania Nascimento, Noemí Tomsen, Saray Acedo, Cristina Campos-Alcantara, Christopher Cabib, Marta Alvarez-Larruy, Pere Clavé

**Affiliations:** 1 Gastrointestinal Physiology Laboratory, Department of Surgery, Hospital de Mataró, Universitat Autónoma de Barcelona, 08304 Mataró, Spain; wdo@csdm.cat (W.N.); ntomsen@csdm.cat (N.T.); malvarezla@csdm.cat (M.A.-L.); 2 Centro de Investigación Biomédica en Red de Enfermedades Hepáticas y Digestivas (CIBERehd), Instituto de Salud Carlos III, Madrid 28029, Spain; ccabibitalia@yahoo.it; 3 Institut de Neurociències, Universitat Autònoma de Barcelona, 08193 Barcelona, Spain; 4 Facultat de Biologia, University of Barcelona, 08028 Barcelona, Spain; acedosaray@gmail.com (S.A.); cristinacamposub@gmail.com (C.C.-A.); 5 Department of Neurology, Hospital de Mataró, 08304 Mataró, Spain

**Keywords:** disorders, spontaneous swallowing frequency, capsaicin, TRPV Cation Channels, stroke, aging

## Abstract

Spontaneous swallowing contributes to airway protection and depends on the activation of brainstem reflex circuits in the central pattern generator (CPG). We studied the effect of age and gender on spontaneous swallowing frequency (SSF) in healthy volunteers and assessed basal SSF and TRPV1 stimulation effect on SSF in patients with post-stroke oropharyngeal dysphagia (OD). The effect of age and gender on SSF was examined on 141 healthy adult volunteers (HV) divided into three groups: GI—18–39 yr, GII—40–59 yr, and GIII—>60 yr. OD was assessed by the Volume–Viscosity Swallowing Test (VVST). The effect of sensory stimulation with capsaicin 10^−5^ M (TRPV1 agonist) was evaluated in 17 patients with post-stroke OD, using the SSF. SSF was recorded in all participants during 10 min using surface electromyography (sEMG) of the suprahyoid muscles and an omnidirectional accelerometer placed over the cricothyroid cartilage. SSF was significantly reduced in GII (0.73 ± 0.50 swallows/min; *p* = 0.0385) and GIII (0.50 ± 0.31 swallows/min; *p* < 0.0001) compared to GI (1.03 ± 0.62 swallows/min), and there was a moderate significant correlation between age and SFF (r = −0.3810; *p* < 0.0001). No effect of gender on SSF was observed. Capsaicin caused a strong and significant increase in SSF after the TRPV1 stimulation when comparing to basal condition (pre-capsaicin: 0.41 ± 0.32 swallows/min vs post-capsaicin: 0.81 ± 0.51 swallow/min; *p* = 0.0003). OD in patients with post-stroke OD and acute stimulation with TRPV1 agonists caused a significant increase in SSF, further suggesting the potential role of pharmacological stimulation of sensory pathways as a therapeutic strategy for CPG activation in patients with OD.

## 1. Introduction

Spontaneous swallowing contributes to airway protection and a reduced spontaneous swallowing frequency (SSF) can lead to increased pharyngeal secretions and aspiration [1]. The neurophysiology of spontaneous swallows falls within the normal functionality of a brainstem reflex arc situated in the central pattern generator (CPG). The activity of CPG can be modified by inputs from cortical and subcortical areas of the brain [2,3]. Previous studies with functional magnetic resonance imaging have shown that spontaneous saliva and water swallows activate the precentral motor and post-central somatosensory cortical areas [4,5], suggesting that the neural control of swallowing not only occurs in the brainstem but also in the cortex [6]. Under physiological circumstances, brainstem reflex circuits may receive excitatory or inhibitory descending nerve impulses from supratentorial structures that modulate final motor response. Excitatory supranuclear influences are proportional to brain excitability. This has been well documented in neurophysiological studies on the blink reflexes in patients with stroke or multiple sclerosis [7,8], and it is hypothesized that the nerve control of spontaneous swallowing could be similar. The SSF in healthy volunteers is 1.32 swallows/min [9], which decreases to approximately 0.6 swallows/min in older people without swallowing impairments [10]. Patients with acute stroke and oropharyngeal dysphagia (OD) showed a significant reduction in SSF (0.23 swallows/min) compared to post-stroke patients without OD (0.56 swallows/min), suggesting that a SSF ≤ 0.40 swallows/min could indicate swallowing impairments [11,12] and identify dysphagia with psychometric properties equal or superior to clinical screening protocols [12]. In addition, a correlation between SSF and salivary substance P (SP) concentration has been described; a low concentration of SP in saliva predicts a significant reduction in SFF and a higher incidence of pneumonia in patients with acute stroke [13].

The human oropharynx is highly innervated by the sensory branches of the cranial nerves V, VII, IX, and X [14]. The TRPV1 receptor has been located in the plasma membrane of the epithelial cells and nociceptive Aδ fibers located in the submucosa near the basal lamina of the human oropharyngeal mucosa [15]. This receptor can be activated with several endogenous and exogenous stimuli, such as pH and temperature changes and natural agonists like capsaicin, facilitating the transmission of sensory inputs through the afferent pathway by the release of several neuropeptides such as SP and CGRP. The role of these neuropeptides in swallowing is still unknown, but it is hypothesized that it could be similar to that described for the cough reflex. In this case, the neuropeptide SP sensitizes the mechanoreceptor fibers and improves the transmission of sensory inputs from the peripheral nerves to the brainstem [16]. In addition, our group found that those patients with OD as a consequence of aging or stroke had lengthened latency and reduced amplitude of the pharyngeal sensory evoked potentials (PSEP) characteristic peaks, in addition to a loss of symmetry of the PSEPs and their cortical representation in patients with chronic post-stroke dysphagia. Our results suggest that impaired conduction and integration of the sensory inputs could affect the efferent pathway, leading to an impaired oropharyngeal swallow response [17,18].

Several studies have demonstrated the therapeutic effect of capsaicin on the biomechanics and neurophysiology of the swallow response in patients with OD. Acute (or single dose) stimulation with capsaicin at 150 μM reduces the prevalence of safety and efficacy impairments of swallowing, and strongly improves the time to laryngeal vestibule closure (LVC) and to upper esophageal sphincter opening (UESO) [19,20]. Similar results have been observed when patients were treated three times a day during 10 days with a lower dose (10 μM). In this case, not only a reduction in time to LVC and PAS score were found, but also a faster and more intense neurophysiological response and significant changes in brain activation, as well as a positive correlation between the variation of N1 peak latency of PSEPs and improved time to LVC [21,22]. Regarding the effect on neuropeptide secretion, an increase in the concentration of salivary SP and CGRP was observed after TRPV1 stimulation with capsaicin [23,24]. Taken all together, these findings suggest that the TRPV1 agonist capsaicin could be used as a potential treatment to improve SSF.

The aims of this proof-of-concept study are (1) to assess the effect of age and gender on SSF in healthy volunteers, (2) to examine whether patients with post-stroke OD have lower SSF, and (3) to assess the effect of TRPV1 stimulation with capsaicin on SSF in patients with post-stroke OD. 

## 2. Materials and Methods 

### 2.1. Study Population

This study has two branches: (1) an observational one-day study to assess the effect of age and sex on spontaneous swallow frequency (SSF) in healthy volunteers and (2) an interventional study to assess the effect of TRPV1 stimulation with capsaicin at 10 μM on SSF in post-stroke dysphagia (PSD) patients. The study protocol was approved by the Ethical Committee of the Hospital de Mataró (11/17) and performed according to the rules of the Declaration of Helsinki. All the participants signed the informed consent.

#### 2.1.1. Healthy Volunteers

A total of 141 healthy volunteers (18–90 years old) were recruited from the community and divided in three groups according to age: 18–39 years (GI), 40–59 years (GII), and ≥60 years (GIII). The inclusion criteria were to be ≥18 years old without previous diagnosis of swallowing disorders or using any medication that could influence the saliva flow (such as antidepressants, antipsychotics, or anticholinergics). The exclusion criteria were head and neck surgery/radiotherapy or neurological disorders including neurodegenerative diseases. 

#### 2.1.2. Stroke Patients

Seventeen stroke patients were recruited through the Neurology Department of the Hospital de Mataró. All patients had signs of impaired swallowing safety according to the Volume–Viscosity Swallowing Test [25]. The exclusion criteria were to have a life expectancy of less than three months, OD diagnosis previous to the stroke episode, or OD associated a pathology other than stroke.

### 2.2. Experimental Design 

**Figure 1:**
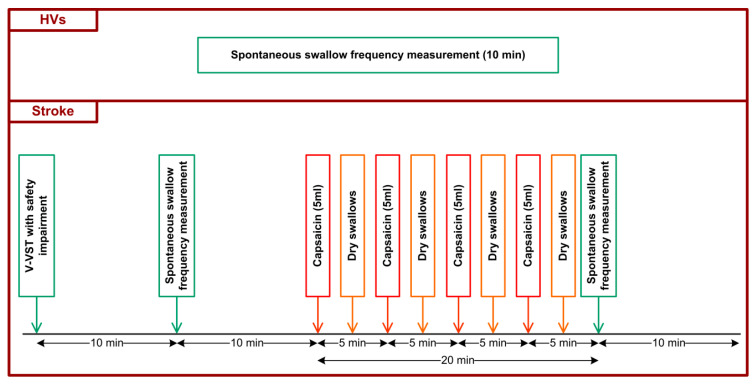
Study design of the two populations included in the study: HV at the top, post-stroke patients at the bottom.

For the observational study, SSF was measured during 10 min in HV and PSD patients. All participants were requested to avoid body and head movements and talking during the experiment. For the study on the effect of capsaicin on SSF, PSD patients received TRPV1 stimulation treatment using a 10 μM capsaicin solution administered orally (four bolus of 5 mL) after the first 10-min SSF recording. In the intervals between capsaicin intakes, patients were requested to make dry swallows (two to four). A second 10-min SSF recording was made following the capsaicin treatment (Figure 1).

### 2.3. Volume–Viscosity Swallowing Test (V-VST)

Stroke patients were screened with the V-VST, as previously described [25]. The swallowing function was assessed while swallowing boluses of 5, 10, or 20 mL of liquid (<50 mPa·s), nectar (98.61 ± 3.78 mPa·s), or pudding (4539.50 ± 530.93 mPa·s). Nectar and pudding were obtained by adding 4.5 and 9 g of Resource ThickenUp (Nestle, Barcelona, Spain), respectively, to 100 mL of water. Only those patients that presented signs of safety impairment (cough, voice changes, and/or a decrease of ≥3% of oxygen saturation) were included.

### 2.4. Spontaneous Swallow Frequency Recordings (SSF)

SSF was measured with surface neck electromyography (EMG) and accelerometry. After cleaning the skin with alcohol, EMG electrodes were positioned over the suprahyoid muscles, while an omnidirectional accelerometer was placed over the cricothyroid cartilage (Supplementary Figure S1). This omnidirectional accelerometer assesses acceleration in multiple directions, but is more sensitive to movement in the vertical plane (Puyau, 2004). SSF was recorded for 10 min. All recordings were analyzed offline using the AcqKnowledge software (BIOPAC Systems Inc., Goleta, CA, USA), which displays a visual trace of the EMG, the complete EMG, and the accelerometer signal. A spontaneous swallow was considered when the signal was registered by both EMG and the accelerometer (Supplementary Figure S2). The spontaneous swallowing rate was calculated as spontaneous swallows per minute. In addition, the following EMG metrics were analyzed for each swallow: amplitude (distance from highest to lowest peak of the EMG signal expressed in volts), duration, delta T (∆T, time from start to end in seconds), and area under the curve (AUC, amplitude and ∆T integral, in volts·seconds).

### 2.5. Intervention: Effect of Oropharyngeal Sensory Stimulation with TRPV1 Agonists

To evaluate the effect of TRPV1 stimulation on SSF, participants were given four 5 mL boluses of 10^−5^ M capsaicin (Spectrum chemical MFG Corp, New Brunswick, NJ, USA), 5 min apart. Between each bolus, patients were requested to perform 2–4 dry swallows depending on their tolerance.

### 2.6. Data Analysis and Statistical Methods

Continuous data were described as mean ± standard deviation (SD) and categorical data as absolute or relative frequencies. Continuous data were analyzed by one-way ANOVA and Dunn’s multiple comparison post-test (age effect), unpaired *t*-test (gender × age effect), or paired *t*-test (pre vs. post TRPV1 stimulation). Categorical data were compared by Fisher’s exact test. The correlation between age and SSF was determined with Spearman’s correlation coefficient. A non-parametric test was performed when appropriate (non-Gaussian data). In order to know if the variables age and sex (independent variables) had a direct effect on SSF (dependent variable), a multiple linear regression test was performed. Statistically significant differences were considered when *p*-value < 0.05. Statistical analysis was performed using the software GraphPad Prism 9 (GraphPad Software, San Diego, CA, USA).

## 3. Results

### 3.1. Study Population

A total of 141 healthy volunteers were included in the exploratory study and divided as follows: 50 in GI (28.08 ± 5.84 years, 54.00% women), 49 in GII (50.08 ± 4.71 years, 51.02% women), and 42 in GIII (63.38 ± 7.92 years, 47.62% women). 

Regarding the PSD patients, a total of 17 PSD patients (74.94 ± 11.43 years, 41.2% women) were included in the study, 11 following an acute stroke and six patients with a chronic stroke (≥3 month from the stroke onset). Mean Rankin was f 3.35 ± 0.93, Barthel index was 69.41 ± 27.43, and NIHSS was 6.59 ± 5.23. The main type of stroke was ischemic (90.10%), and most of them were located in the left hemisphere (43.75%). According to the Oxford classification, 18.75% had a total anterior circulation infarct, 43.75% partial anterior circulation infarct, 6.25% lacunar infarct, and 31.25% had posterior circulation infarct. V-VST results showed that 100% of PSD patients had signs of impaired safety of swallow and 82.35% also had efficacy impairment signs. Liquid was the most unsafe viscosity (70.59%), followed by nectar (29.41%), and pudding was the safest one (5.88%) (*p* < 0.0001). Regarding the efficacy impairments, pudding viscosity presented the highest rate of pharyngeal residue (76.47%) when compared to nectar (58.82%) and liquid (25%) (*p* = 0.01). Indications for VFS were personalized according to the evolution and clinical setting, and mean PAS score was 4.00 ± 2.58.

### 3.2. Basal SSF in HV and PSD Patients

**Figure 2:**
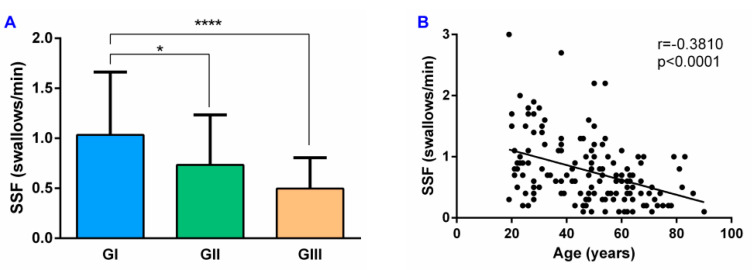
SSF and its correlation with age in HV: (**A**) SSF results by age groups; (**B**) correlation between SSF and age. SSF: spontaneous swallowing frequency; GI: 18–39 years age group; GII: 40–59 years age group; GIII: ≥60 years age group; * *p* < 0.05; **** *p* < 0.0001.

The mean SSF in all HV was 0.8 ± 0.5 swallows/min. Analyzing the results according to the age of the participants, SSF in the GI was 1.03 ± 0.62 swallows/min and was significantly reduced in GII (0.73 ± 0.50 swallows/min; *p* = 0.0385) and GIII (0.50 ± 0.31 swallows/min; *p* < 0.0001) (Figure 2A). In addition, there was a moderate significant negative correlation between age and SSF (r = −0.3810, *p* < 0.0001) (Figure 2B).

**Figure 3:**
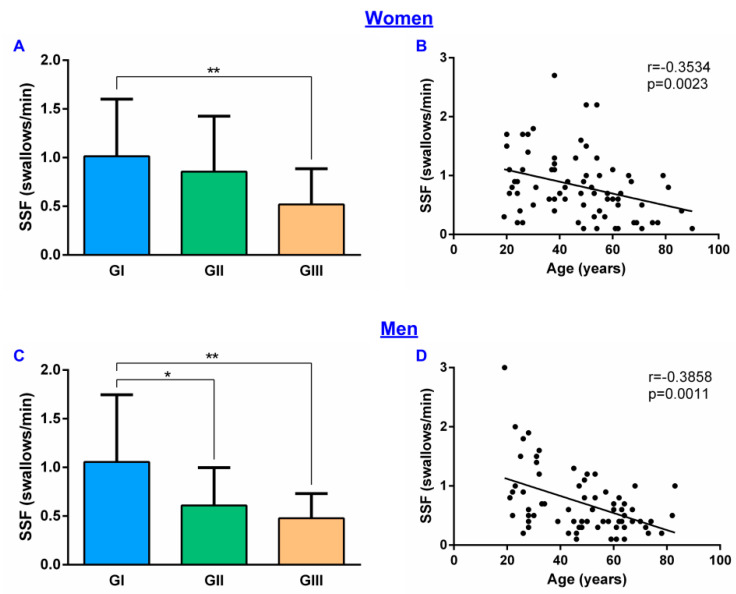
Gender effect on SSF: (**A**) SSF results by age groups in women; (**B**) correlation between SSF and age in women; (**C**) SSF results by age groups in men; (**D**) correlation between SSF and age in men. SSF: spontaneous swallowing frequency; GI: 18–39 years age group; GII: 40–59 years age group; GIII: ≥60 years age group; * *p* < 0.05; ** *p* < 0.01.

Regarding the effect of gender, the mean SSF in women was 0.82 ± 0.55 swallows/min and in men 0.72 ± 0.53 swallows/min (*p* = 0.1846). When comparing these results between age groups, in women there were significant differences between GI (1.01 ± 0.57 swallows/min) and GIII (0.52 ± 0.36 swallows/min; *p* = 0.0067) groups but not with GII (0.86 ± 0.56 swallows/min) (Figure 3A). In men, however, there was a significant reduction in GII (0.61 ± 0.38 swallows/min; *p* = 0.0345) and GIII (0.48 ± 0.25 swallows/min; *p* = 0.0029) when compared to GI (1.06 ± 0.67 swallows/min) (Figure 3C). A negative correlation between SSF and age was also found in women (r = −0.3534, *p* = 0.0023) (Figure 3B) and men (r = −0.3858, *p* = 0.0011) (Figure 3D). However, according to the multiple linear regression test, our data show that SSF is only affected by age (F(1, 138) = 24.55; *p* < 0.0001) but not by gender (F(1, 138) = 1.347; *p* = 0.2479).

Finally, PSD patients showed an SSF of 0.41 ± 0.32 swallows/min without significant differences when compared to the same age group in HV (GIII) (*p* = 0.3112).

### 3.3. Basal EMG Metrics

**Figure 4:**
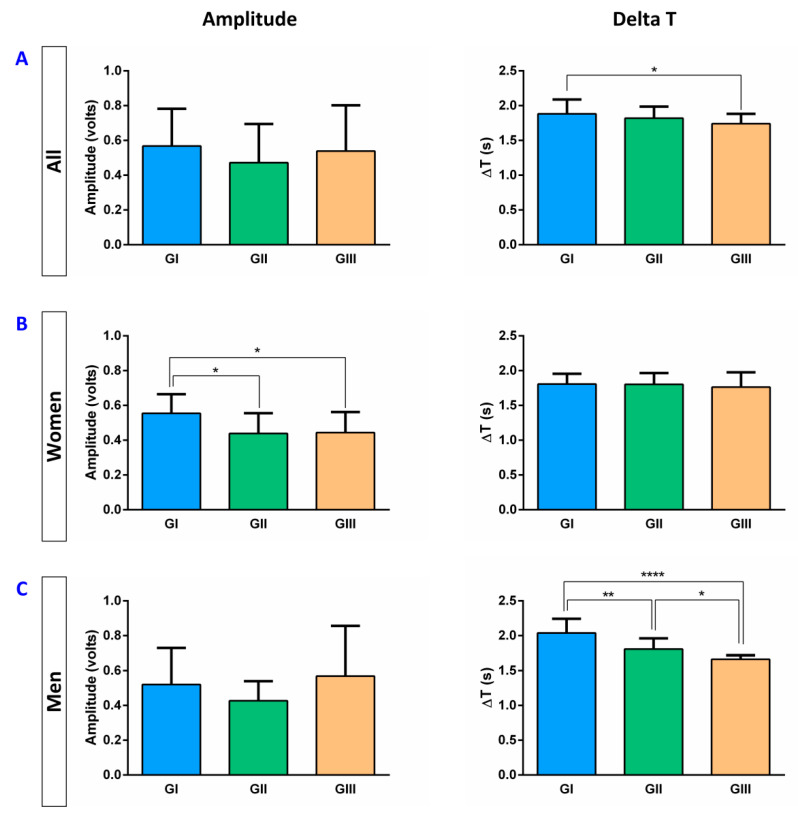
EMG metrics (Amplitude and Delta T) in all HV (**A**), women (**B**), and men (**C**). ∆T: Delta T; s: seconds; GI: 18–39 years group; GII: 40–59 years group; GIII: ≥60 years group; * *p* < 0.05; ** *p* < 0.01; **** *p* < 0.0001.

In HV, the mean amplitude was 0.70 ± 0.61 volts, the mean delta T, 1.82 ± 0.39 s, and the mean AUC, 0.26 ± 0.37 volts·seconds. Taking into account the three age groups, we found a significant reduction only in the duration of the EMG signal (delta T) that was reduced by age between GI (1.88 ± 0.20 s) and GIII (1.74 ± 0.14 s; *p* = 0.0178) (Figure 4A). Regarding the effect of gender, significant differences between men and women were observed. Women showed a reduction in the amplitude (GII: 0.44 ± 0.11 volts, *p* = 0.0158; GIII: 0.46 ± 0.13 volts, *p* = 0.0500; vs. GI: 0.55 ± 0.11 volts) with age (Figure 4B) while in men, a reduction in delta T (GI: 2.04 ± 0.19; GII: 1.81 ± 0.15 s, *p* = 0.0071 (vs. GI); GIII: 1.66 ± 0.05, *p* < 0.0001 (vs. GI) and *p* = 0.0439 (vs. GII)) was observed (Figure 4C). No significant differences were observed for AUC.

**Figure 5:**
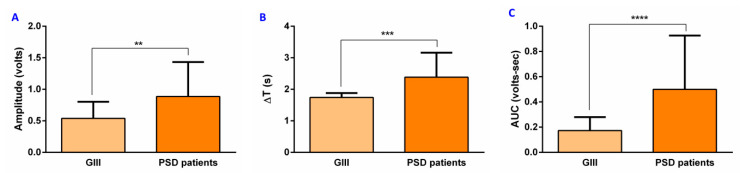
EMG metrics in PSD patients and GIII group: (**A**) amplitude, (**B**) Delta T, and (**C**) AUC. ∆T: Delta T; AUC: area under the curve; s: seconds; GIII: ≥60 years group; PSD: post-stroke dysphagia; ** *p* < 0.01; *** *p* < 0.001; **** *p* < 0.0001.

We also found that PSD patients presented an increased amplitude (0.89 ± 0.55 volts, *p* = 0.0029) (Figure 5A), delta T (2.38 ± 0.78 s, *p* = 0.0004) (Figure 5B), and AUC (0.50 ± 0.43 volts-seconds, *p* < 0.0001) (Figure 5C) when compared to HV in GIII group with similar age.

### 3.4. Effect of TRPV1 Stimulation on SSF and EMG Metrics in PSD

**Figure 6:**
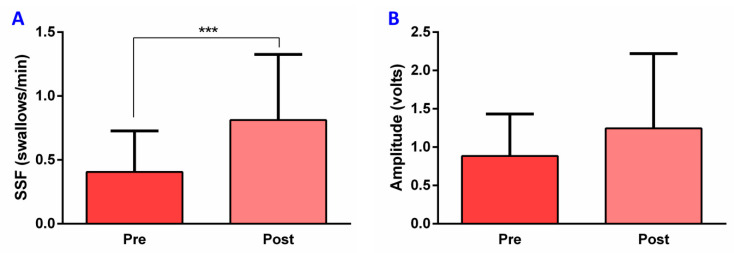
Capsaicin effect on SSF and EMG metrics in post-stroke dysphagia patients: (**A**) SFF, (**B**) Amplitude. SSF: spontaneous swallowing frequency; s: seconds; pre: pre-treatment; post: post-treatment; *** *p* < 0.001.

**Figure 7:**
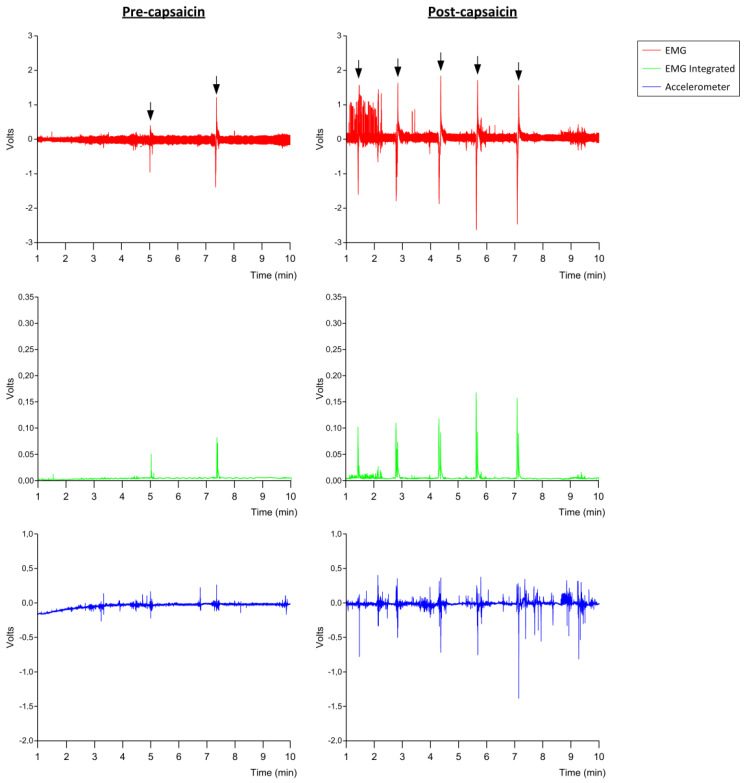
Pre- and post-capsaicin stimulation SSF registration. Red line represents the electromyography (EMG) signal; green line represents the EMG integrated signal; blue line represents the accelerometer movement; black arrows point to each swallow registered.

After TRPV1 stimulation with capsaicin (10^−5^ M), PSD patients showed a significant increase in SSF (0.81 ± 0.51 swallows/min, *p* = 0.0003) compared to the frequency registered before treatment (0.41 ± 0.32 swallows/min) (Figure 6A and Figure 7). However, no significant changes in the other EMG metrics were observed: amplitude pre-treatment, 0.89 ± 0.55 volts, vs. post-treatment, 1.24 ± 0.98 volts (Figure 6B), *p* = 0.1503; delta T pre-treatment, 2.38 ± 0.78 s, vs. post-treatment, 2.30 ± 0.66 s, *p* = 0.7831; AUC pre-treatment, 0.50 ± 0.43 volts·second, vs. post-treatment, 0.59 ± 0.57 volts·second, *p* = 0.8400.

## 4. Discussion

The aims of this proof-of-concept study were to describe the effect of age and gender on SSF and its associated metrics, to assess whether patients with post-stroke OD had impaired SSF and to assess the potential therapeutic effect of oropharyngeal sensory stimulation with a TRPV1 agonist such as capsaicin on SSF. In addition, we also explored the behavior of some metrics associated with SSF (amplitude, duration, AUC) in both experimental situations. We found SFF was significantly reduced by age but not by gender in HV. We also observed a trend towards reduced amplitude and duration of SSF with age. Interestingly, post-stroke dysphagic patients showed increased basal amplitude, duration, and AUC when compared to older healthy people. Acute stimulation with capsaicin caused a significant double-fold increase in SSF, further suggesting the potential role of sensory stimulation as a therapeutic strategy for CPG activation in dysphagic patients without effect on amplitude or duration. 

The first result of the study was a significant reduction of SSF with age, a 29.13% reduction in GII group, and 51.47% in GIII group in comparison to GI group. We also observed that there was a significant negative correlation between age and SSF. These results concur with those previously described [9,10]. In addition, there was a reduction of the duration of each swallow in the GIII group compared to GI. Spontaneous swallowing is rhythmic motor behavior, such as breathing and sucking [26], and its motor control depends on both the peripheral and central nervous systems. Although there is no evidence of how aging impairs spontaneous swallowing, in previous studies we found that older people show a decrease in pharyngeal sensitivity, and significant alteration of the pharyngeal sensory evoked potentials, much more pronounced in older patients with OD [17]. This impairment in the sensory function is related to a reduction in the small myelinated fibers of the superior laryngeal nerve [27] and peripheral neurodegeneration of the oropharyngeal mechanoreceptors fibers, alterations that correlate with impaired mastication and swallowing function in an aging animal model [28]. We previously found that this impairment in the afferent pathway of the neurophysiological swallowing response was associated with the impaired biomechanics of oropharyngeal swallow response (OSR). Several studies from our group have shown that older people, especially those with OD, present an altered OSR, especially delayed time to LVC, which leads to an increase in the prevalence of penetrations and aspirations [29]. In this study, we found a similar effect of age on the reduction of SSF paralleling the decrease in pharyngeal sensory function and the delay in time to LVC. When we analyzed the data by age and gender, we observed that women showed a significant reduction only in SSF in GIII in comparison with the GI group. On the other hand, men showed a significant reduction in both GII and GIII groups when compared to the GI group. When we performed a multiple linear regression analysis, however, we found that SSF was only affected by the variable age but not by gender. 

Regarding the EMG metrics, the amplitude, delta T, and AUC of each swallow were evaluated. The amplitude represents the maximal force contraction; the delta T, the duration of the muscular contraction; and the AUC, the integral of the amplitude and the delta T of the suprahyoid muscle contraction, showing the overall muscular effort during each swallow. We observed a reduction in the amplitude in women with age, while in men it was the duration of each swallow that was affected by age. These results agree with what has been previously described [30,31,32] and could be explained by the involutive changes in these muscles that are observed with aging as a consequence of different causes such as malnutrition or sarcopenia [33]. Taken together, we hypothesize this reduction in SSF with age in healthy volunteers parallels the decrease in the pharyngeal sensory function of these persons.

Regarding the SSF in patients with post-stroke OD, no significant differences were found when compared to the GIII HV group (HV of the same age). In contrast to what was previously described [11,12], we did not observe significant differences on SSF between HV and post-stroke patients with OD due to the reduced number of patients included in the present study and also to the combination of acute and chronic patients. In contrast, we observed significant changes in the EMG metric, with a significant increase in the amplitude, the duration, and the AUC of the muscular contraction associated with each swallow. The increased duration of each swallow could be related to the prolonged neurophysiological and biomechanical response (time from GPJO-LVO) of swallowing reported in post-stroke patients [34]. In addition to a delayed oropharyngeal and prolonged swallow response, these patients showed a loss of symmetry of the pharyngeal sensory evoked potentials and their cortical representation [18], and a reduced and delayed pharyngeal motor evoked potentials [35]. About the increase in amplitude, few studies have related the increase in this variable in older patients with OD to the loss of adipose tissue, specifically in the submental region, as fat attenuates the EMG signals [32,36]. Another interpretation could be that these patients with post-stroke OD require a stronger muscular effort to elicit a spontaneous swallow.

The hypothesis that the neural control of spontaneous swallowing depends on the CPG and could be similar to that described for the blink reflex [2,3,7,8], leads to peripheral neurostimulation strategies as a possible treatment to improve SSF. One of the most used chemical strategies to improve swallow response is capsaicin stimulation. This natural agonist activates TRPV1 receptors, which are widely located in the epithelial cells and nerve endings of the human oropharynx and could be activated by endogenous or exogenous agonists [15]. After acute treatment with capsaicin 10^−5^ M, post-stroke patients showed a significant increase in SSF without any major changes in the EMG metrics, probably due to the small sample. In a previous study in healthy subjects, significant changes in pharyngeal and UES function were assessed by manometric and EMG metrics after an acute treatment with capsaicinoids at the same concentration used in our study [37]. Our group also has demonstrated the therapeutic effect of capsaicin at different concentrations and acute/subacute administration. Regarding the acute (single dose) treatment, we observed an improvement in time to LVC and UESO, reduction in the prevalence of oral and pharyngeal residue, and increased cortical excitability at a concentration of 150 μM but not at 10^−5^ M [19,22,38]. The low dose showed better results when patients received the treatment over 10 days, three times a day. In this case, patients not only showed improvements in biomechanics but also in neurophysiology, inducing a faster and more intense response by shortening the latency, and increasing the amplitude of the pharyngeal sensory evoked potential peaks. In addition, we described a significant correlation between the improvement of N1 peak latency and the improvement of time to LVC, suggesting that neuroplasticity processes were being induced that resulted in improved OSR [21,22]. With this evidence, we can conclude that capsaicin would be inducing greater conduction of stimuli through the afferent pathway to the CPG, resulting in an enhancement of the SSF.

This proof-of-concept study had some limitations. First of all, the sample size of patients with post-stroke OD is quite small, combined patients in the acute and chronic state, and swallowing function was only assessed with V-VST. We are working on another prospective study where a larger sample of patients with an acute stroke and OD will be included. In addition, a group of acute post-stroke patients without OD will also be included. Further studies are needed to explore the SSF in OD patients to know if it could be used as a screening tool. Finally, it would be interesting to study if there are changes in the concentration of salivary SP after capsaicin treatment and if it is related to the improvement observed in SSF. Additionally, the duration of the improvement in SSF following TRPV1 stimulation with capsaicin should be measured.

## 5. Conclusions

Spontaneous swallowing frequency ranged around one swallow per minute in healthy young volunteers, which was significantly affected by age but not by gender. Post-stroke patients with OD showed no differences in SSF compared with HV of similar age but presented higher amplitude and longer duration of each spontaneous swallowing. Acute stimulation with capsaicin results in a significant increase in SSF, suggesting the involvement of oropharyngeal TRPV1 receptors on the swallowing reflex control and further supporting a potential pharmacological strategy to treat post-stroke patients with OD with these pharmacological compounds. 

## Data Availability

The data presented in this study are available within the article and their supplementary material.

## Supplementary Materials

The following are available online at https://www.mdpi.com/2075-4418/11/3/461/s1: Figure S1. Placement of the electrodes (blue arrow) and accelerometer (red arrow). Figure S2. Example of a swallowing signal. The EMG signal is shown in red, the EMG integrated in green and the accelerometer in blue. EMG: electromyography.
