# Supplementary material for: Effect of Aging, Gender and Sensory Stimulation of TRPV1 Receptors with Capsaicin on Spontaneous Swallowing Frequency in Patients with Oropharyngeal Dysphagia: A Proof-of-Concept Study
Source: Diagnostics (Basel). 2021 Mar 7;11(3):461. doi: 10.3390/diagnostics11030461 (PMC7999082; doi:10.3390/diagnostics11030461)
Supplement: Supplementary file 1 [file diagnostics-11-00461-s001.pdf]

diagnostics

Article

Effect of Aging, Gender and Sensory Stimulation of TRPV1 Receptors with Capsaicin on Spontaneous Swallowing Frequency in Patients with Oropharyngeal Dysphagia: A Proof-of-Concept Study

Supplementary Materials

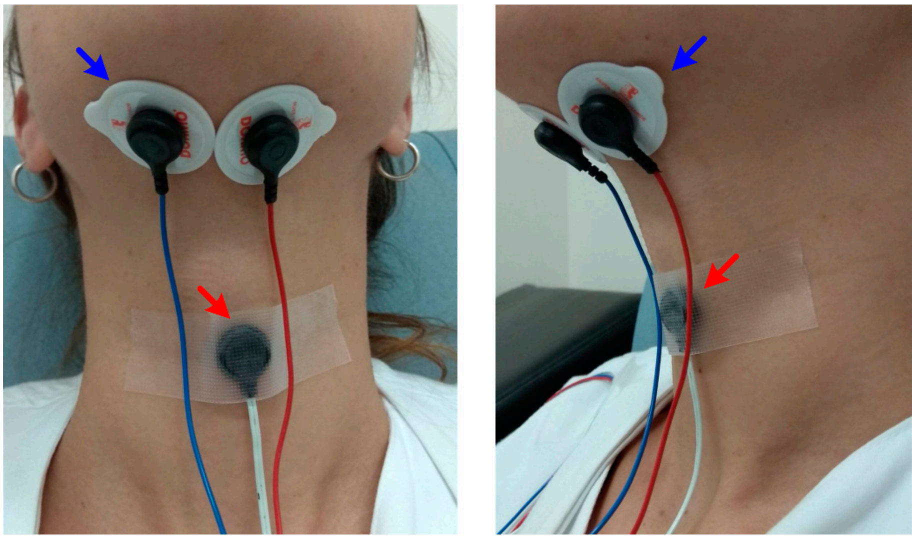

Figure S1. Placement of the electrodes (blue arrow) and accelerometer (red arrow).

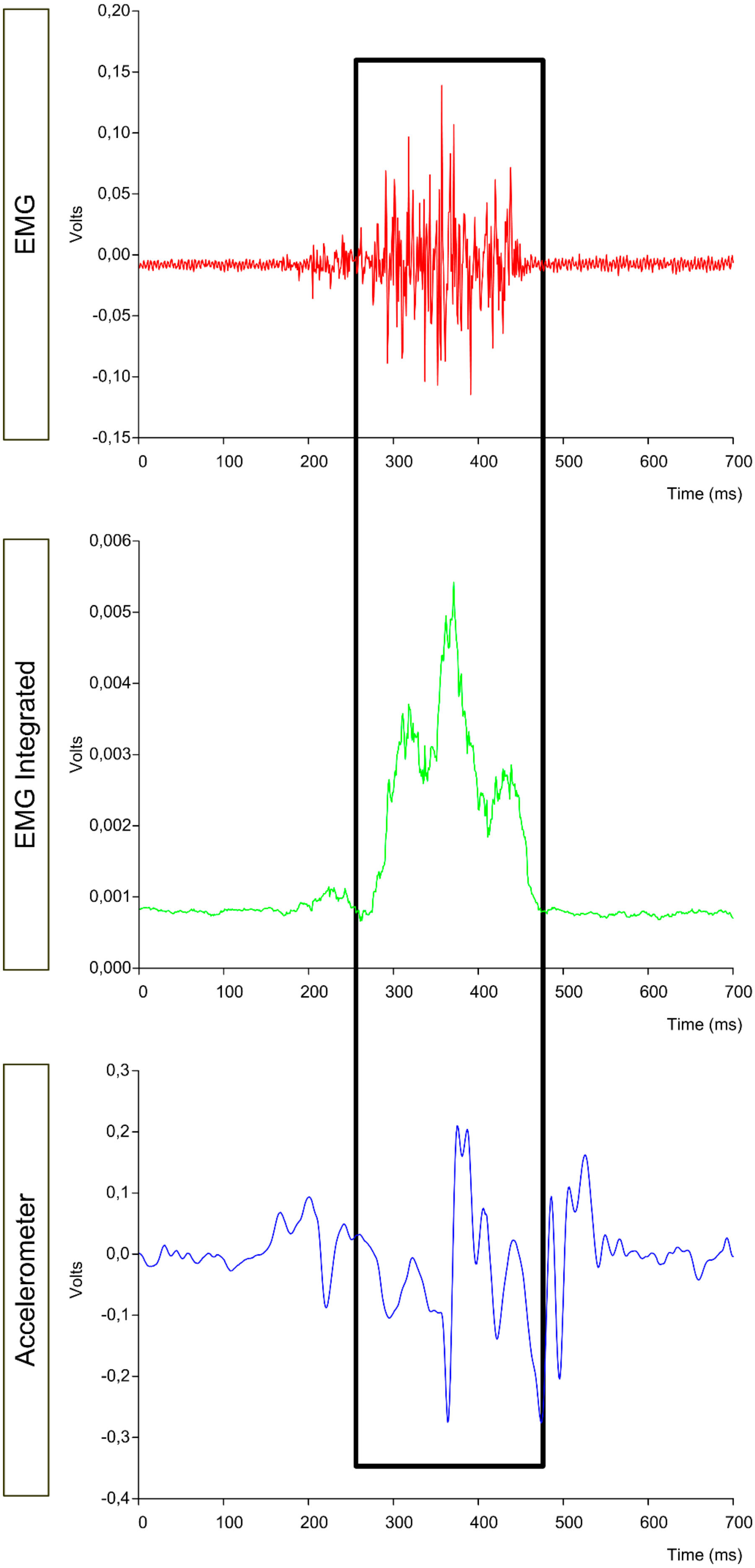
EMG
Volts
0,20
0,15
0,10
0,05
0,00
-0,05
-0,10
-0,15
0
100
200
300
400
500
600
700
Time (ms)
EMG Integrated
Volts
0,006
0,005
0,004
0,003
0,002
0,001
0,000
Time (ms)
Accelerometer
Volts
0,3
0,2
0,1
0,0
-0,1
-0,2
-0,3
-0,4
Time (ms)

Figure S2. Example of a swallowing signal. The EMG signal is shown in red, the EMG integrated in green and the accelerometer in blue. EMG: electromyography.
